# Harmful Algae in a Changing World: Where Did You Come from and Where Are We Going

**DOI:** 10.3390/toxins18050196

**Published:** 2026-04-23

**Authors:** Katia Comte

**Affiliations:** UMR 7245 Molécules de Communication et Adaptation des Microorganismes (MCAM), Muséum National d’Histoire Naturelle, CNRS, CP 39, 57 rue Cuvier, 75005 Paris, France; katia.comte@mnhn.fr

Aquatic environments, whether freshwater, brackish, or marine, are increasingly disrupted, in terms of frequency, extent, geographic distribution, and duration, by the massive, worldwide proliferation of harmful and/or nuisance algae, the so-called Harmful algal blooms (HABs), which are a global phenomenon that poses a major threat to human and animal health and ecosystems. While the majority of freshwater HABs are caused by cyanobacteria (cHAB), the marine bloom-forming species (approximately 300 species) are microalgae with detrimental effects, 80 of which are toxin-producing species belonging to eukaryotic flagellates such as Dinoflagellates and Raphidophytes (responsible for red-tides), as well as other groups including Diatoms, Haptophytes, Dictyophytes, and prokaryotic Cyanobacteria.

Not all species responsible for HABs act in the same way, nor do they all pose the same hazards to ecosystems and their biocenoses. A wide variety of secondary metabolites and modes of action (most of which are still poorly understood) exist within harmful species, leading to the classification of HABs into three types: the first includes “true” toxic species that produce various toxins that affect the entire food web (from protists to higher trophic levels, including humans) and cause various episodes of poisoning. The second type is defined by “non-toxic” species, whose large biomass alone is sufficient to induce serious damage and mass mortality of aquatic organisms through a drastic drop in dissolved oxygen concentration (transition from hypoxia to anoxia). The third type is represented by “non-toxin-producing” species exhibiting cytotoxic or ichthyotoxic properties, *via* multiple and not yet fully elucidated biochemical and physical mechanisms, such as the lytic properties of the membranes of other organisms (for example, the gills of fish), the production of reactive oxygen species (ROS), the harmful effects of certain polyunsaturated fatty acids (PUFAs), and the production of dense mucus. These “ichthyotoxic” microalgae represent approximately 70 to 80 species of phytoplankton, including dinoflagellates, raphidophytes, dictyophytes, and marine haptophytes from around the world.

There is a current consensus regarding the main causes of the global expansion of HABs worldwide, which is the excessive and accelerating eutrophication process by anthropogenic pressure in all water bodies. The ever-increasing input of nutrients (primarily phosphorus and nitrogen) from agricultural land, livestock farming, and their discharges into coastal waters poses a major global challenge that is difficult to contain due to its persistent storage resulting from massive accumulation in sediments over decades in aquatic environments.

Another major issue is climate change, with global warming projected at 1.7 to 4.9 °C between 1900 and 2100. In addition to the temperature rise in all aquatic systems, other direct and indirect consequences will appear, such as sea level rise (from 0.1 to 0.9 m in 2100–IPCC, 2001), ocean acidification due to increased CO_2_, thermohaline, and/or water column stratification on a global scale, as well as extreme weather events (floods and droughts).

The extent to which these abiotic changes, identified as key stressors for environments, will affect the biodiversity, the expansion of the HABs species (and therefore their toxicity), and the functioning of water systems, including ecosystem services and economic resources (aquaculture, fisheries, tourism, etc.), constitutes a crucial issue for centuries to come on a global scale.

This Special Issue was open to the scientific community working on harmful algal blooms in freshwater and marine environments. It addresses the differences between past and present approaches to studying these HABs species (“where do we come from?”), and innovative approaches related to new methods of investigation that enable the early detection of species responsible for HABs or the measurement of their toxin concentrations in water samples (“where are we going?”).

In the first article, Wejnerowski et al. [1] analyzed the cyanobacterial community structure in an eutrophic lake in Poland, and compared the diversity of species between summer and winter seasons with a multiphasic approach, including classical microscopy observations, molecular analysis (genes implying in the toxin synthesis), and analytical techniques (HPLC-DAD, LC/MS, and ELISA) for two cyanotoxins detection (microcystin: MC and cylindrospermopsin: CYN) from water samples and isolated strains from the field. They found a large dominance of cyanobacteria on the water surface (around 94% in summer and 70% in winter), mainly represented by Oscillatoriales species, including *Planktothrix* and *Limnothrix* genera in summer, and dominated by *Limnothrix* in winter. They confirmed that the *Planktothrix* strains were MC-producing species, while no other toxins were detected by analytical methods, with the exception of the *cyrJ* gene in the water samples. Interestingly, they observed a shift within the cyanobacterial community: the year-round dominance of *P. agardhii*, noted in previous studies (2006–2008) in this lake, gave way to a permanent bloom of *Limnothrix redekei,* recently observed in many European lakes. This study provided a new confirmation that eutrophic temperate lakes are "bloom-friendly" for potentially toxic cyanobacteria throughout the year, thus highlighting the need for regular eco-phyco-toxicological monitoring of freshwater systems.

The following article, by Agasild et al. [2], also used the *mcyE* reference gene to detect microcystin-producing cyanobacterial strains, thus exploring a new avenue of research based on the transfer of these MCs within zooplankton communities and their impact on the food web. To date, few studies have examined the ingestion of these microcystins by different zooplankton taxa or the differences in ingestion capacity according to their feeding modes (grazers, filter feeders), using samples collected in situ during toxic blooms. This study provided original data from a eutrophic lake in Estonia, where *Microcystis* dominated the water body. They detected and quantified the *mcyE* gene by qPCR in the gut contents of crustaceans collected in situ, and they found that all guts contained *mcyE,* with significant variations between grazers, including a higher abundance in cyclopoid copepods than in cladocerans. The *mcyE* gene proved to be the preferred tool here, because it is present in a single copy per genome, allowing for an accurate assessment of *Microcystis* cell abundance in each grazing crustacean. They demonstrated that the transfer of toxic cells into the food web is primarily determined by the dynamics of specific and top grazers (omnivorous copepods being the predominant consumers compared to the generalist feeders like daphnia), rather than by the abundance of toxic cyanobacterial strains in natural aquatic environments.

The interactions between toxic species and the food web, in terms of detection and/or bioaccumulation, were also addressed in the article by Litaker et al. [3] using a “hybrid” socio-toxicological approach. This approach combined the analysis of historical data dating back to 1948 concerning a traditional belief among Native Alaskan populations (that consuming a butter clam was safe if it had been prepared beforehand and its siphon removed), with a rigorous evaluation of different protocols and their level of safety, based on the detection of the phycotoxin STX (saxitonin) in each tissue (using HPLC coupled with FLD). This marine toxin was produced by the dinoflagellate *Alexandrium catenella*, which frequently bloomed in Alaskan waters during the summer months, while tending to disappear during the winter.

By analyzing the STX content in all supposedly "edible" tissues, they unequivocally refuted the traditional belief that simple cleaning was sufficient to reliably reduce STX concentration and render the clam “safe” for consumption, particularly during the summer when values reaching 1000 µg STX eq. 100 g of tissue (capable of causing severe symptoms) were recorded. The presence of STX was also observed in winter, well after the disappearance of the *A. catenella* bloom, highlighting the ongoing risk of shellfish contamination by this neurotoxin and underscoring the lack of any means to predict when and where highly toxic shellfish will be harvested, which can have moderate-to-severe public health consequences.

While this study examined the STX concentrations from the common dinoflagellate *Alexandrium* sp and assessed the actual toxicity of ingesting shellfish contaminated with PSP, the other two articles [4,5] focused on the detection of potentially toxic marine species in two anthropized locations, a port in Tahiti [4] and an aquaculture area (in New Zealand) [5] by the use of a fairly recent technique: environmental DNA in situ (eDNA).

Fernandez et al. [4] used an alternative molecular method, environmental DNA (eDNA) metabarcoding of water samples, to assess the diversity of potentially toxic marine species in the port of Papeete (Tahiti), which could be a hotspot for a new large-scale distribution of species responsible for HABs. They identified a total of 21 potentially toxic species, including three directly associated with mass fish mortality events in this area and 18 other species known to produce a wide range of phycotoxins, including ciguatoxins (PSPs), other CFP toxins, and domoic acid (ASPs type, produced by diatoms). They found that the eDNA of *Gambierdiscus* sp. coincided with several ciguatera outbreaks in Tahiti between 2018 and 2024 and demonstrated that the majority of HABs detected by metabarcoding were currently present throughout the port area, from shallow to deep waters, potentially posing significant risks to seafood consumption and, consequently, to human health. Although this study did not confirm the actual presence of toxins in port waters (due to a lack of additional toxicological data), and therefore could not formally demonstrate a direct causal relationship, these results suggest the usefulness of eDNA analysis for the early detection of HABs and its potential as a promising tool for the routine monitoring and prevention of human risks associated with new alien contamination by various non-native species in these transit environments.

The study by Biessy et al. [5] was conducted in a New Zealand aquaculture area where shellfish farms are highly exposed to blooms of *Alexandrium pacificum,* a PSP-producing species that could impair biological resources and lead to economic losses for the region. The authors investigated biological interactions during and after *Alexandrium* blooms using high-throughput sequencing on prokaryotic and eukaryotic communities over two consecutive summers. They found significant changes in the composition of microbial communities, dominated by Rhodobacterales and Flavobacteriales during the *Alexandrium* bloom, and a high abundance of Pelagibacteriales during post-bloom. A shift towards the dominance of Syndiniales and Gymnodiniales eukaryotes was noted during the decline of the bloom, suggesting their possible ecological role in regulating and terminating this phase, as Syndiniales are known for their parasitic interactions (leading to the death of their hosts). Concurrently, they found that copepods, particularly the *Paracalanus*, dominated the water column during the bloom period, indicating their high capacity to feed on toxic dinoflagellates and tolerate their toxicity levels. They emphasized the need to investigate parasite groups as well as grazing zooplankton as potential bioagents for mitigating harmful algal blooms (HABs) in aquaculture and marine environments. Their study makes an innovative contribution by combining metabarcoding and bloom ecology to reveal biotic interactions and key ecological factors, with a view to developing targeted management strategies to mitigate future HABs.

The last three articles [6,7,8] address *in vivo* experimental studies focused on the development of new molecular tools for the specific detection of toxins directly in producing cells or in water samples. They exploit the chemical nature of DNA structure to develop fluorescence-based methods enabling the specific detection of NRPS (non-ribosomal peptide synthetases) involved in the synthesis of microcystins (MCs) and anabaenopeptides APS [7,8] or of oligonucleotides specific to the marine phycotoxins [6].

The study by Al Tabban et al. [6] described a fluorescent approach for the simultaneous detection of six marine toxins (including MCs, anatoxin-a, STX, OA, brevetoxin (BTX), and cylindrospermopsin (CYN)) *in vivo,* on strains, and in water samples. This original method relies on a duplex-to-complex structure-switching approach using an aptamer sequence (used as a bioreceptor) specific to each targeted toxin, which is labeled with a fluorescent dye and combined with their corresponding cDNA, conjugated with a fluorescent quencher. In the absence of toxin in the water sample, the aptamer–cDNA complex is formed, and the fluorescence is quenched (=inhibited). If the toxin is present in the sample, the aptamer binds to its specific target (in this case, the toxin) and releases the cDNA (the aptamer’s affinity for its target being higher than its affinity for the cDNA used). An aptamer–toxin complex then forms, and fluorescence is restored, its intensity being correlated with the toxin concentration. High sensitivity was observed for the detection of all six toxins, with good selectivity for each aptamer and no cross-reactivity. These results demonstrate that the aptamer-based technique could be an excellent alternative to traditional methods (immunoassays and analytical techniques such as LC/MS) for the in situ and simultaneous detection of various toxins, which is crucial for ensuring water safety.

The last two articles by Kurmayer et al. [7,8] described an innovative method based on the incorporation (i.e., “clickable”) of non-natural amino acids into the adenylation domain of the NRPS of two common freshwater cyanotoxins: microcystins (MCs) in *Microcystis* cells and anabaenopeptins (APS) in *Planktothrix agardhii*. The juxtaposition of a fluorochrome to these amino acids provided chemo-selective labeling, allowing the clickable cyanopeptide (i.e., chemically modified cyanopeptide) to be localized in the cells and to track the intracellular dynamics by analytical chemistry and high-resolution imaging (laser-scanning confocal microscopy). The first study [7] confirmed a quantitative relationship between clickable modified cyanopeptide content and the signal intensity resulting from chemo-selective labeling (during maximum growth) throughout time-lapse build-up or decline experiments. The second study [8] focused more specifically on the time-lapse experiment, using the pulse feeding of three non-natural amino acids to observe the synthesis and/or decay of chemically modified MC and APS in *Microcystis* and *Planktothrix* cells under maximum growth rate conditions. The authors demonstrated a rapid increase in clickable MC and APS content, linked to the growth rate, and a steady decay of clickable MC and APS through dilution due to the growth, rather than a regulated or induced release during the synthesis process. This highlights a new method for tracking toxin synthesis and its intracellular dynamics, and paves the way for promising management strategies for preventing risks to water safety and public health through the early detection of toxin-producing cells in water bodies.

To conclude, this Special Issue presents new data and innovative approaches to better understand the distribution and presence of harmful algal blooms in all aquatic systems, as well as their potential expansion in the face of global surface water warming and the resulting unforeseen environmental changes.

While these articles offer new perspectives on the early and large-scale detection of HAB species (including the presence of their respective toxins) in marine and freshwater ecosystems, using both classical and exploratory molecular tools, they all focus on toxin-producing species. This underscores the need to complement broader research efforts on other classes of HABs, including “non-toxic” species associated with animal mortality or health impairments, for which these latest innovative fluorescent-toxin-complex techniques will not be applicable. This need is all the more fundamental as an acceleration in the widespread distribution and frequency of these “nuisance” algae is predicted through flash blooms due to global warming and a rapidly changing world.
